# FunFOLD5: combining localised templates and structural homology for accurate predictions of biologically relevant protein–ligand interactions

**DOI:** 10.1093/bioinformatics/btag762

**Published:** 2026-10-10

**Authors:** Recep Adiyaman, Liam James McGuffin

**Affiliations:** School of Biological Sciences, University of Reading, Reading RG6 6DH, United Kingdom; School of Biological Sciences, University of Reading, Reading RG6 6DH, United Kingdom

## Abstract

**Motivation:**

A central challenge in protein–ligand modelling is to predict not only the presence of an interaction but also the correct ligand identity, binding-site location, and biologically relevant bound conformation. While recent deep learning approaches such as AlphaFold3 and Boltz-2 have demonstrated impressive accuracy in modelling protein–ligand complexes, these methods typically require prior knowledge of the ligand, focus on a single dominant binding pose, and rely heavily on large training datasets. Template-based methods offer a complementary solution by leveraging experimentally resolved protein–ligand complexes to transfer both binding-site architecture and ligand chemical identity, enabling function inference even for uncharacterised or engineered proteins. However, conventional template-based methods are limited by their dependence on global fold similarity, which can miss local binding features critical for ligand specificity. Localised template search strategies may overcome this limitation by capturing conserved pocket-level interactions, enabling more accurate and functionally relevant ligand-binding predictions across diverse protein families.

**Results:**

We present FunFOLD5, a template-based method for predicting protein–ligand binding sites. Whereas its predecessor FunFOLD4 relied on global fold similarity to identify templates, FunFOLD5 combines local and global searches, integrating rapid structural template retrieval with a local targeted protocol to improve interaction accuracy. The pipeline combines Foldseek-based template searching, curated ligand annotations from BioLiP2, and ligand superposition followed by spatial clustering to identify associated ligands and consensus binding sites. Benchmarking on blind targets from CASP15 and CASP16 demonstrates robust performance across a wide range of biologically relevant ligands, including nucleotides, metal ions, cofactors, and small metabolites. FunFOLD5 achieved high-accuracy predictions for 81.2% of ligand instances (56 of 69). Importantly, the local targeted protocol consistently improved ligand identification, residue-level recall, and binding-pose accuracy.

**Availability and implementation:**

FunFOLD5 is freely available as a Dockerised pipeline at https://hub.docker.com/r/radiyaman/funfold5_template, providing a scalable and reproducible framework for large-scale protein–ligand interaction analysis and functional annotation.

## 1 Introduction

A protein’s function is defined by its interactions with other biomolecules, particularly small-molecule ligands, which play a central role in regulating biological activity and underpin key applications in drug discovery, enzyme engineering, and functional annotation (Roche *et al.* 2011, 2012, 2013, Burley 2021). Despite advances in structural biology, the experimental determination of protein–ligand complexes remains costly, time-consuming, and often infeasible due to factors such as protein instability, membrane association, or transient complex formation (Roche *et al.* 2011, Burley 2021). Moreover, ligands with low binding affinity are often lost during purification processes, posing significant challenges for the experimental determination of their three-dimensional (3D) conformations and precise binding locations. In such cases, computational approaches provide an efficient and scalable alternative for predicting ligand-binding sites and inferring protein function, particularly when high-resolution structural data are unavailable or incomplete. Sequence- and structure-based *in silico* tools enable scalable, cost-effective protein–ligand interaction prediction for functional annotation and drug discovery.

Protein–ligand interaction prediction methods fall into two broad categories: sequence-based and structure-based approaches. Sequence-based methods leverage evolutionary conservation and functional motifs (Capra and Singh 2007) but lack 3D context, making them insufficient for capturing conformational flexibility, non-conserved binding sites, or novel folds (Lichtarge *et al.* 1996, Wass *et al.* 2010, 2012). Consequently, these methods perform poorly for proteins with low sequence similarity.

Structure-based methods utilise 3D protein structures, derived either from experimental techniques or computational prediction, to identify and characterise ligand-binding interactions. These methods are typically grouped into four major categories: molecular docking, molecular dynamics simulations, machine learning-based approaches, and template-based methods. Each of these relies on structural information to varying degrees and offers distinct advantages and limitations in terms of accuracy, interpretability, and data requirements (Dhakal *et al.* 2022, Utgés and Barton 2024, Wang 2024).

Molecular docking predicts ligand poses within a protein’s binding site using scoring functions to estimate binding affinity (Morris *et al.* 2009). Although efficient, docking typically assumes rigid or semi-rigid protein conformations and requires prior knowledge of the ligand and binding site, thereby limiting its applicability. Molecular dynamics simulations capture conformational flexibility and solvent effects, but are computationally intensive and depend on an initial binding hypothesis. Together, these limitations underscore the need for approaches that operate without prior ligand or site information (Dhakal *et al.* 2022, Utgés and Barton 2024, Wang 2024).

In recent years, deep learning has revolutionised protein–ligand interaction prediction. Early in this progression, RoseTTAFold-All-Atom (Krishna *et al.* 2024) advanced atomic-level modelling of protein structures and complexes with ligands, laying the foundation for all-atom AI-driven approaches. Chai Discovery introduced a multimodal attention-based framework capable of predicting diverse biomolecular complexes, including protein–ligand and protein–nucleic acid interactions. Notably, Chai-1 (Chai Discovery Team *et al.* 2024) can operate in a single-sequence mode without requiring multiple sequence alignments, making it highly adaptable for novel or poorly characterised targets. Subsequently, AlphaFold3 (Abramson *et al.* 2024) extended the success of AlphaFold2 through a generative diffusion architecture that jointly models proteins, small molecules, nucleic acids, and ions. AlphaFold3 currently sets the benchmark in protein–ligand complex prediction, demonstrating state-of-the-art accuracy on benchmark datasets and offering particular promise for structure-based drug discovery. Most recently, Boltz-2 (Passaro *et al.* 2025) introduced a substantive advance by combining physical energy principles with generative neural networks to co-predict ligand-bound structures and, for the first time in the field, directly estimate binding affinities, marking a significant step forward in protein–ligand modelling.

Machine learning has advanced protein–ligand interaction prediction through explicit ligand modelling; however, dependence on prior ligand knowledge, biases in training data, and assumptions of protein rigidity limit performance on novel ligands, unconventional folds, and engineered proteins. Moreover, many models prioritise a single binding site and fail to capture ligand flexibility, induced fit, allostery, and dynamic binding, highlighting the need for bias-aware, dynamics-informed approaches that support discovery-driven functional annotation (Dhakal *et al.* 2022, Utgés and Barton 2024, Wang 2024).

Template-based approaches exploit structural homology to transfer binding-site information from experimentally determined protein–ligand complexes to query proteins, allowing accurate inference of binding regions and plausible ligands even when the chemotype is unknown (Brylinski and Skolnick 2010, Wass *et al.* 2010, Roche *et al.* 2011, Yang *et al.* 2013b). Unlike docking and machine learning approaches, template-based methods leverage ligand-bound experimental structures to provide interpretable, biologically grounded predictions when the structural signal is strong. This makes them particularly effective for large-scale structural genomics, the annotation of engineered proteins, and exploratory work in protein design and synthetic biology. In practice, targets are aligned to known complexes from curated resources such as the Protein Data Bank (PDB; Protein Data Bank 1971) and BioLiP (Yang *et al.* 2013a), and ligand and residue annotations are transferred based on conserved 3D geometry. Established tools such as FINDSITE (Skolnick and Brylinski 2009), 3DLigandSite (Wass *et al.* 2010), COACH-D (Wu *et al.* 2018), and FunFOLD (Roche *et al.* 2011) exemplify this paradigm.

FunFOLD5 is a template-based method for predicting protein–ligand binding sites, building on previous versions of the FunFOLD pipeline. Earlier versions such as FunFOLD4 depended only on global fold similarity, whereas FunFOLD5 integrates both local and global search, giving weight to binding-region resemblance as well as overall structure. By superposing pocket geometries and their coordinating residues across numerous ligand-bound templates, FunFOLD5 forms a reliable consensus for each site, suppresses noise from unrelated folds, and favours the ligand chemistries that best match the observed microenvironment. This pocket-centred strategy enables the identification of secondary or allosteric sites and supports function assignment in uncharacterised or engineered proteins, where global fold similarity may be weak or misleading. As a result, FunFOLD5 provides a practical advantage over conventional template-based methods by improving both sensitivity and interpretability.

## 2 Materials and methods

### 2.1 The FunFOLD pipeline and ligand annotation

In its public web server implementation, FunFOLD3 (Roche *et al.* 2011, Brackenridge and McGuffin 2021), the pipeline integrates structural quality assessment from FunFOLDQA (Roche *et al.* 2012) to provide confidence estimates for predicted binding sites. FunFOLD takes as input the top-ranked 3D models generated by the IntFOLD server (McGuffin *et al.* 2019, 2023), together with the list of identified structural templates.

To ensure biological relevance, FunFOLD integrates information from the BioLiP database (Yang *et al.* 2013a), which combines computational and manual curation of biologically meaningful protein–ligand complexes. BioLiP provides detailed annotations for each entry, including binding residues, Enzyme Commission (EC) numbers (Matsuta *et al.* 2013), and Gene Ontology (GO) terms (Harris *et al.* 2004). These data enable FunFOLD to distinguish biologically relevant ligands from those that are artefacts of crystallisation or non-functional binding events. Only templates containing BioLiP-validated ligands and achieving TM-scores (Zhang and Skolnick 2005) > 0.4 are retained for subsequent analysis (Roche *et al.* 2011, 2013).

FunFOLD has been extensively benchmarked in earlier Critical Assessment of Structure Prediction (CASP) experiments (Soro and Tramontano 2005, López *et al.* 2009, Roche *et al.* 2011, Gallo Cassarino *et al.* 2014). Since its first participation in CASP8, FunFOLD has consistently ranked among the top-performing methods for ligand-binding site prediction. In CASP10, although only 13 of 97 targets were found to be biologically relevant, FunFOLD demonstrated strong performance and interpretability across tested targets (Gallo Cassarino *et al.* 2014). In CASP15, the protein–ligand category was relaunched to include ligand interactions irrespective of biological relevance, and FunFOLD5 was benchmarked in CASP15 and CASP16, with predictions submitted by our group during each of the respective blind prediction seasons (Robin *et al.* 2023, Tosstorff *et al.* 2026).

FunFOLD5 introduces major improvements over previous versions by integrating global and local template searches with Foldseek (van Kempen *et al.* 2024) for structural template retrieval and upgrading to the BioLiP2 (Zhang *et al.* 2024) database for ligand annotation. While FunFOLD4 identifies templates through global search alone, FunFOLD5 uses both global and local searches to select templates more accurately. FunFOLD5 requires a single input 3D structure from IntFOLD, AlphaFold, or any other predictor. Template retrieval is accelerated by Foldseek, which replaces computationally expensive modelling and alignment steps with an ultra-fast 3Di alphabet-based structural search, enabling structure comparisons against the complete PDB within seconds. In FunFOLD5, this same TM-score >0.4 threshold is applied to Foldseek-derived templates to maintain consistency with the original FunFOLD methodology. This approach preserves the rigour of the established FunFOLD selection criteria while taking advantage of Foldseek’s internal statistical ranking, thereby streamlining the workflow and reducing computational overhead.

The BioLiP2 database (Zhang *et al.* 2024) replaces the original BioLiP (Yang *et al.* 2013a) as the source of biologically relevant ligand-bound templates. It expands coverage of experimentally validated protein–ligand complexes, improves structural classification, and provides enhanced functional annotation, including EC numbers and GO terms, while filtering crystallographic artefacts and non-biological ligands to ensure the transfer of biological interactions. Together, Foldseek and BioLiP2 enable FunFOLD5 to deliver faster and broader ligand-binding site predictions while preserving accuracy and interpretability grounded in experimentally observed structures, with substantial gains in scalability and computational efficiency.

To enhance both sensitivity and precision, the FunFOLD5 pipeline employs a dual-stage architecture for predicting protein–ligand binding sites. As illustrated in Fig. 1, this process integrates the global fold similarity approach of FunFOLD4 with a focused analysis of local binding-site characteristics. In the first stage, the full 3D query model is analysed with Foldseek to identify relevant templates via an ultra-fast search of the PDB using both 3Di and TM-align modes. Retained templates are then structurally superimposed onto the query model using TM-align (Zhang and Skolnick 2005). BioLiP2 provides curated data on experimentally validated protein–ligand complexes, including ligand identities and binding residues. After ligand transfer, FunFOLD5 clusters ligand atoms by spatial proximity, identifies the most supported cluster as the primary binding site, and assigns residues within 0.5 Å van der Waals contact as binding residues, yielding the initial global prediction as shown in Fig. 1c. A ligand atom and a protein atom are counted as in contact when their separation does not exceed the sum of their element-specific van der Waals radii plus 0.5 Å, so the threshold scales with atom size rather than imposing a single fixed distance on every pair; this is the definition used in the CASP ligand-binding assessments and in previous FunFOLD versions (Roche *et al.* 2011). All candidate binding sites are identified simultaneously within this single global pass, with the number recovered determined by template availability. Each site is then refined sequentially in the local stage, one site at a time, with its own local template list.

**Figure 1:**
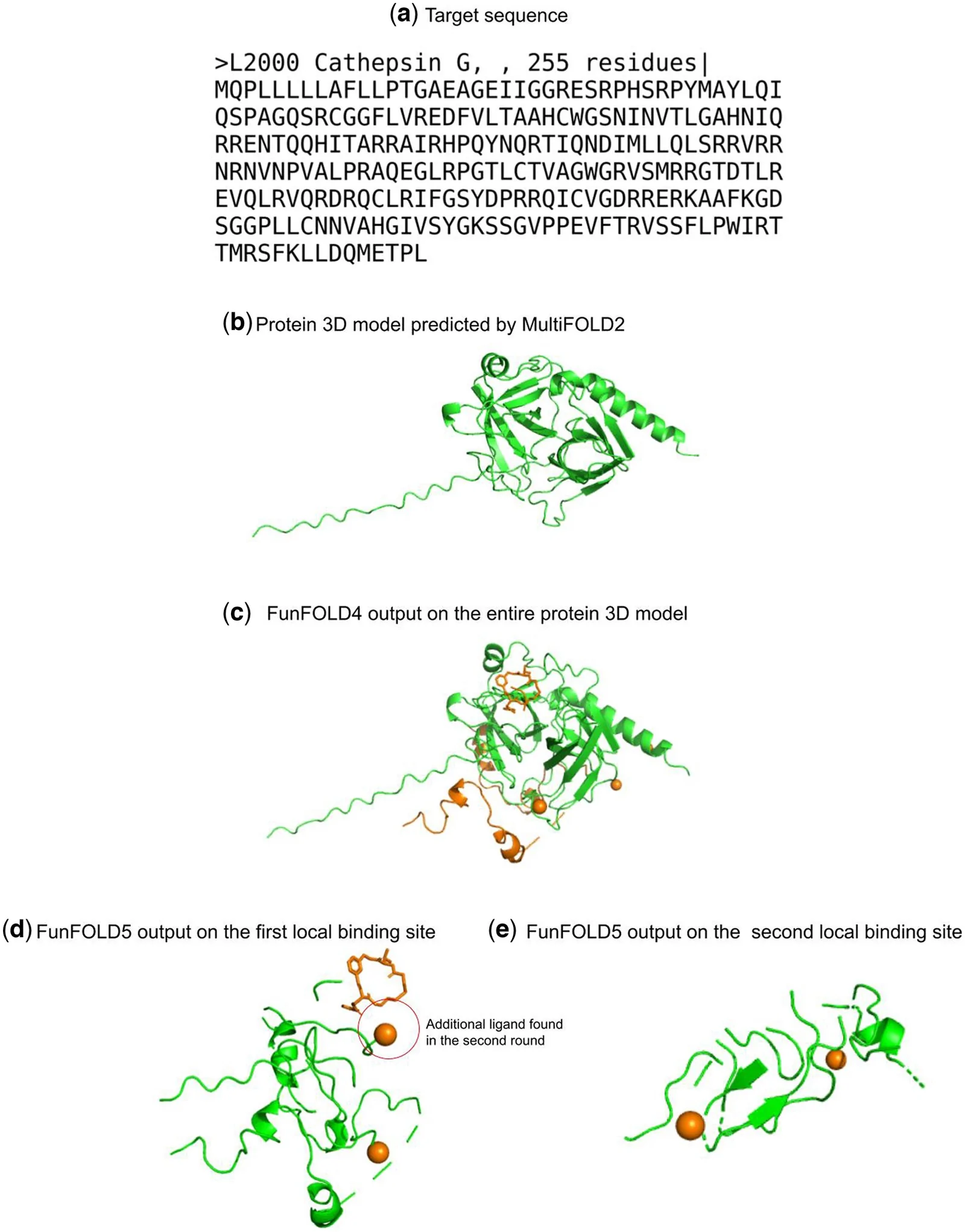
Output from the FunFOLD5 protocol. (a) Protein sequence for the CASP16 target L2000. (b) Predicted 3D model of L2000 generated by MultiFOLD2 (McGuffin *et al.* 2025). (c) FunFOLD4 output for the full 3D model of L2000, highlighting template-derived ligand-binding sites (template ligand coloured orange). (d) The local stage of the FunFOLD5 process focuses on the primary local binding region detected during the first phase of the analysis. (e) Local targeted protocol output focusing on the second local binding site identified in the initial round.

To improve prediction sensitivity and to identify additional or secondary binding sites that may not have been detected in the global search, as in FunFOLD4, FunFOLD5 employs a local targeted protocol. In this stage, a local structural region centred on the binding sites identified in the first round is extracted using PyMOL (DeLano 2002). The extracted region includes the binding residues themselves, together with adjacent loop segments lying within ∼10 Å of the binding site. This local substructure is then re-submitted to Foldseek, but in this case, the search focuses on local binding-region similarity rather than global fold similarity.

Templates were retrieved with the Foldseek web API against pdb100 (iterativesearch enabled), using 3Di-AA mode for the whole-structure search and both 3Di-AA and TM-align for local searches. Retrieved hits were filtered by deposition date and by the TM-score ≥0.4 criterion applied within FunFOLD, which is the retention threshold established in previous versions. This value lies below the TM-score of 0.5 conventionally taken to indicate a shared fold, so that remotely related templates whose binding pockets may still be conserved are retained, while remaining well above the level of ∼0.17 at which structural similarity becomes indistinguishable from random (Xu and Zhang 2010). Local regions comprised residues within 10 Å of the binding site, with binding residues assigned within van der Waals contact plus 0.5 Å. Ligand annotations were taken from BioLiP2 (downloaded 10 April 2024). The CASP15 (2022-04-30) and CASP16 (2024-04-30) deposition cut-offs were applied to both the template hits and the BioLiP2 annotations and Foldseek template lists for each template.

The local binding-site templates obtained from this step are processed through the same FunFOLD5 protocol. Each local template is aligned using TM-align, ligand coordinates from BioLiP2 are superimposed, and ligand atoms are spatially clustered to identify potential protein–ligand interactions. Each local binding site produces its own refined template list, enabling the identification of additional ligands, alternative binding pockets, and ligand poses that were not apparent during the initial global search, as in FunFOLD4 (Fig. 1d and e).

By integrating both global fold-based and local pocket-based searches, FunFOLD5 can identify dominant and secondary ligand-binding regions, thereby improving coverage and enabling the discovery of novel or functionally relevant binding sites that may otherwise remain undetected in a purely global search.

Runtimes were measured across 14 benchmark targets (1,838–13,409 model atoms, 25 binding sites) on a workstation with an Intel Core i9 (14 cores, 20 threads). Template retrieval took ∼2 min regardless of model size, and complete annotation took a median of 33 min per target, or about 11 min per binding site (T1170, two sites: 36 min). Runtime therefore scales with the number of binding sites rather than model size.

## 3 Results and discussion

### 3.1 Blind benchmark performance in CASP15 and CASP16

FunFOLD5 and FunFOLD4 were benchmarked under blind conditions on CASP15 and CASP16 ligand targets. The experimentally observed ligands corresponded to biologically relevant ligands found in the PDB, as summarised in Table 1, available as supplementary data at *Bioinformatics* online. This filtering was necessary because many CASP ligand targets featured pharmaceutical or synthetic ligands which did not correspond to biologically relevant PDB templates. This analysis reflects the template availability at the time of the CASP experiments.

**Table 1:** Comparison of the FunFOLD4 global and FunFOLD5 local targeted predictions against experimentally observed binding residues.

CASP target	Ligand	PDB ID	Observed binding residues	FunFOLD4 (global)			FunFOLD5 (local targeted)			Change	
				Binding residues	BDT	MCC	Binding residues	BDT	MCC	ΔBDT	ΔMCC
T1124	SAH (1)	7ux8	163, 167, 170, 202, 203, 204, 208, 225, 226, 229, 251–254, 268–270, 273	163, 170, 173, 201, 202, 224, 225, 253, 254, 269, 270, 272, 282, 320	0.604	0.479	163, 167, 170, 173, 204, 229, 253, 269, 320	0.422	0.533	−0.182	+0.054
T1124	SAH (2)	7ux8	163, 167, 170, 202–204, 208, 225, 226, 229, 251–254, 268–270, 273	163, 170, 202, 204, 208, 253, 254, 268–270	0.556	0.736	163, 167, 202–204, 208, 225, 229, 253, 254, 268–270	0.722	0.843	+0.167	+0.107
T1127v2	CoA (1)	8xbp	31, 137, 138, 143–146, 148, 149, 177, 180, 181, 184	28, 31, 32, 136–138, 140, 143, 144, 145, 148, 171, 175, 177, 178, 182	0.772	0.542	31, 136, 138, 143–146, 148, 149, 175, 177, 178, 180–182, 184	0.896	0.820	+0.124	+0.278
T1158v4	ATP (1)	8swn	183, 419, 448, 450–453, 480, 1175–1180	446, 447, 448	0.156	0.156	447, 448, 450–453, 480, 592	0.524	0.585	+0.368	+0.429
T1158v4	Mg^2+^ (3)	8swn	452, 480	447, 480	0.596	0.499	452, 480	1.000	1.000	+0.404	+0.501
T1170	ADP (1)	7pbr	5–7, 14, 47, 49–52, 166, 202, 203	13, 14, 47–52, 163, 166, 174, 202	0.824	0.653	6, 7, 13, 14, 48–52, 166, 174, 203	0.882	0.740	+0.058	+0.087
T1170	ADP (2)	7pbr	6, 7, 14, 47–52, 166, 202, 203	7, 13, 14, 46–52, 166, 174, 202, 203, 206	0.870	0.812	6, 7, 13, 14, 46–52, 166, 174, 202, 203, 206	0.878	0.860	+0.008	+0.048
T1170	AGS (1)	7pbr	5–7, 14, 46, 47, 49–52, 156, 166, 203	7, 13, 14, 46–52, 166, 174, 202, 203, 206	0.846	0.703	6, 7, 13, 14, 46–52, 166, 174, 202, 203, 206	0.856	0.751	+0.010	+0.049
T1188	DW0 (1)	8c6z	120, 166, 172, 247, 252	64, 117–119, 123, 125–127, 157–162, 190, 193, 217	0.243	−0.016	154, 156, 215, 216	0.078	−0.008	−0.165	+0.008
H1171	ADP (1)	7pbl	6, 7, 13, 14, 47–52, 166, 202, 203, 206	6, 7, 13, 14, 45–52, 95, 166, 174, 202, 203, 206	0.867	0.876	6, 7, 13, 14, 46–52, 95, 166, 174, 202, 203, 206	0.896	0.903	+0.029	+0.027
H1171	AGS (1)	7pbl	7, 13, 14, 47, 49–52, 166, 202, 203, 206	6, 7, 13, 14, 45–52, 95, 166, 174, 202, 203, 206	0.826	0.808	6, 7, 13, 14, 46–52, 95, 166, 174, 202, 203, 206	0.853	0.833	+0.027	+0.025
H1171	Mg^2+^ (2)	7pbl	51, 203	6, 7, 13, 14, 45–52, 95, 166, 174, 202, 203, 206	0.402	0.325	6, 7, 13, 14, 46–52, 95, 166, 174, 202, 203, 206	0.417	0.335	+0.015	+0.010
H1172	ADP (1)	7pbp	5, 6, 13, 14, 47–52, 113, 166, 203	7, 13, 14, 46–52, 95, 166, 174, 202, 203, 206	0.811	0.679	6, 7, 13, 14, 46–52, 95, 166, 174, 202, 203, 206	0.822	0.727	+0.011	+0.049
H1172	AGS (2)	7pbp	5–7, 13, 14, 46, 47, 49–52, 144, 156, 166, 203	7, 13, 14, 46–52, 95, 166, 174, 202, 203, 206	0.832	0.695	6, 7, 13, 14, 46–52, 95, 166, 174, 202, 203, 206	0.842	0.738	+0.010	+0.043

In CASP15, 28 ligand-bearing targets were released, spanning 153 ligand instances. Of these, 11 targets comprising 67 biologically relevant ligand instances had experimentally observed ligands corresponding to biologically relevant species with available PDB templates and were investigated by FunFOLD5 (T1124, T1127v2, T1158v4, T1170, T1188, H1171v1, H1171v2, H1172v1, H1172v2, H1172v3, and H1172v4); the remaining 17 targets were excluded either because no ligand-bound structure was released, or because no suitable template was available before the CASP15 deadline.

In CASP16, 15 ligand-bearing targets were released, spanning 198 ligand instances, predominantly pharmaceutical or synthetic inhibitors distributed across four industry drug-design datasets (L1000 Chymase, L2000 Cathepsin G, L3000 Autotaxin, and L4000 MPro). Only the Autotaxin set (L3000) yielded biologically relevant ligands with available PDB templates, contributing two investigated ligand instances [L3102, SO_4_^2−^ and L3132, acetate (ACT)]; the remaining 14 targets were excluded. Consequently, template-based benchmarking in CASP16 was restricted to these two biologically relevant ligand instances, reflecting the predominance of synthetic and pharmaceutical chemistry in this round. In contrast, CASP15 comprised ligand classes with clear biological relevance, including nucleotides, cofactors, metal ions, and small metabolites, making it well-suited for assessing FunFOLD5’s intended application.

To support chemically informed evaluation, CASP15 and CASP16 target-bound ligands were grouped into four chemistry-based categories according to their physicochemical properties and structural roles. Rigid ligands, such as sulphate (SO_4_^2−^) in CASP16 target L3102 and magnesium ions (Mg^2+^) in CASP15 targets T1158v4, H1171v1–v2, and H1172v1–v4, bind through fixed coordination geometries that impose strict spatial constraints (Pereira *et al.* 2017, Zheng *et al.* 2017). Nucleotide ligands were observed, including adenosine diphosphate (ADP) in T1170, H1171v1–v2, and H1172v1–v4, and adenosine 5′-[*γ*-thio]triphosphate (AGS) in T1170, H1171v1, and H1172v1–v4 (Saraste *et al.* 1990, Hemmer *et al.* 1997). Canonical ATP binding was observed only in CASP15 target T1158v4. Flexible cofactors, such as coenzyme A (CoA) in T1127v2 and S-adenosylhomocysteine (SAH) in T1124, were also observed (Nguyen *et al.* 2020, Perlinska *et al.* 2020). Small metabolites, including ACT in CASP16 target L3132 and the low-affinity ligand DW0 in CASP15 target T1188, were observed to bind through minimal contacts in solvent-exposed or transient sites, as summarised in Table 1, available as supplementary data at *Bioinformatics* online (Koshland 1958).

Ligand-binding accuracy for CASP15 and CASP16 targets was assessed using LDDT_*pli*_, with predictions classified as high (≥0.4), moderate (0.2–0.4), or low (<0.2) accuracy. Across all benchmarks, FunFOLD5 achieved high-accuracy predictions for 56 of 69 ligands (81.2%), with a further seven cases (10.1%) of moderate accuracy and only six low-accuracy predictions (8.7%). This distribution demonstrates robust and consistent performance, with over four-fifths of ligand-binding sites predicted at high accuracy across chemically diverse ligands and binding site architectures, as shown in Fig. 1, available as supplementary data at *Bioinformatics* online.

FunFOLD5 excelled on CASP16 targets for rigid and small metabolite ligands. For L3102 (sulphate), it ranked top-10 with LDDT_*pli*_ 0.688, RMSD 2.28 Å, LDDT_*pli*_ 0.903, and BB-RMSD 0.324 Å. For L3132 (acetate), it recovered the pocket with LDDT_*pli*_ 0.585, RMSD 3.40 Å, LDDT_*pli*_ 0.921, and BB-RMSD 0.351 Å, as reported in Table 1, available as supplementary data at *Bioinformatics* online.

For CASP15 target T1124, FunFOLD5 predicted two instances of the flexible cofactor SAH (PDB ID 7ux8) with moderate accuracy, yielding LDDT_*pli*_ scores of 0.293 and 0.318 and RMSDs of 9.47 and 8.88 Å, consistent with the ligand’s multi-domain flexibility. For T1127v2, FunFOLD5 predicted two instances of the flexible cofactor CoA (PDB ID 8xbp), achieving high accuracy with LDDT_*pli*_ scores of 0.462 and 0.577 and RMSDs of 6.16 and 6.07 Å (Table 1, available as supplementary data at *Bioinformatics* online).

For T1158v4 (PDB ID: 8swn), FunFOLD5 predicted two ATP and two Mg^2+^ sites. The ATP site prediction showed moderate-to-high accuracy (LDDT_*pli*_ 0.200/0.472, RMSD 9.07/5.77 Å), with stable adenine placement but flexible triphosphate tails. The Mg^2+^ site predictions were highly accurate (LDDT_*pli*_ 0.550/0.839, RMSD 3.35/1.99 Å), with one ranked top-10, demonstrating effective modelling of rigid metal-binding sites (Table 1, available as supplementary data at *Bioinformatics* online).

For T1170 (PDB ID: 7pbr), FunFOLD5 predicted two ADP and four AGS sites. The ADP site predictions were highly accurate (LDDT_*pli*_ 0.894/0.881, RMSD 0.81/0.85 Å), reflecting precise nucleotide-binding motif placement. The AGS site predictions also reached high accuracy (LDDT_*pli*_ 0.691, 0.541, 0.754, 0.828; RMSD 1.29–3.23 Å), with one ranked fourth overall, demonstrating robust recognition of conserved phosphate- and adenine-binding geometries (Table 1, available as supplementary data at *Bioinformatics* online).

For targets H1172v1–v4 (PDB ID: 7pbp), which share the same subunit as T1170, FunFOLD5 showed similarly strong performance. Out of the 13 ADP, 11 AGS, and 11 Mg^2+^ sites, the ADP site predictions ranged from moderate to high accuracy (LDDT_*pli*_ 0.407–0.905; RMSD 0.77–4.89 Å), with H1172v2 and H1172v4 achieving multiple top-1 and top-5 rankings, reflecting consistent recognition of conserved nucleotide-binding motifs. The AGS site predictions were uniformly high accuracy (LDDT_*pli*_ 0.719–0.850; RMSD 1.15–2.11 Å), with top-1 placements in H1172v2/v4, confirming precise placement of phosphate and adenine moieties. The Mg^2+^ site prediction performance varied by subunit: low in H1172v1 (LDDT_*pli*_ ≤0.083, RMSD >8 Å), consistently high in H1172v2–v3 (LDDT_*pli*_ 0.700–0.833; RMSD 2.73–3.51 Å), and partially high in H1172v4 (LDDT_*pli*_ 0.306–0.792), highlighting accurate modelling of rigid metal cofactors when structural constraints are well-defined (Table 1, available as supplementary data at *Bioinformatics* online).

For T1188 (PDB ID: 8c6z), FunFOLD5 predicted two DW0 (water) sites with high accuracy (LDDT_*pli*_ 0.426/0.601; RMSD 4.65/6.39 Å). Although not top-10 ranked, these results demonstrate successful localisation of biologically plausible hydration sites despite diffuse, weakly defined binding geometries (Table 1, available as supplementary data at *Bioinformatics* online).

Nucleotides represented the strongest ligand class in terms of the predictive performance of FunFOLD5. ADP and AGS together comprised nearly half of all ligands, with virtually all predictions in the high-quality tier, reflecting near-perfect localisation of conserved adenine-binding environments (Fig. 2 and Fig. 1, available as supplementary data at *Bioinformatics* online). This performance is driven by strong evolutionary conservation and abundant structural templates. ATP, despite its flexible triphosphate chain, was consistently placed in the correct binding region, with roughly half of the predictions high quality and the remainder moderate, demonstrating robust performance in dynamic binding sites (Fig. 2 and Fig. 1, available as supplementary data at *Bioinformatics* online). Rigid ligands also showed strong performance. Mg^2+^ ions were predicted with high accuracy in roughly half of all cases, while lower-tier predictions were largely confined to solvent-exposed or weakly coordinated sites with intrinsically high positional variability. This trend indicates that FunFOLD5 accuracy is driven by local structural definition rather than ligand identity alone. Sulphate (SO_4_^2−^), though less frequent, was consistently predicted at high accuracy in well-defined anion-binding pockets, highlighting robust modelling of rigid ligands with simple, reproducible geometries.

**Figure 2:**
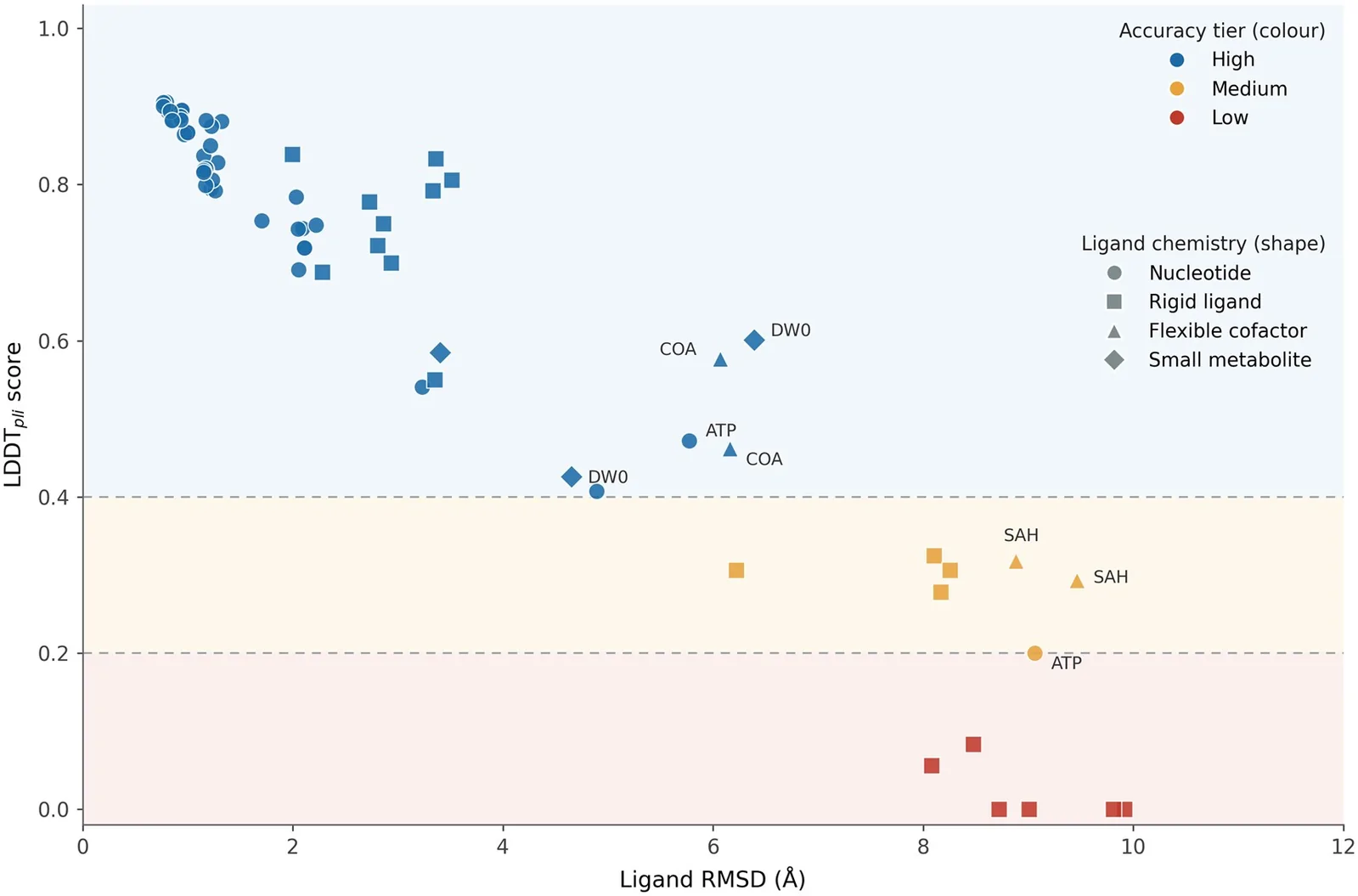
FunFOLD5 ligand-binding prediction accuracy across ligand types (RMSD versus LDDT_*pli*_) across the CASP15 and CASP16 benchmark (69 ligand instances). Each point is one ligand instance, positioned by ligand RMSD against LDDT_*pli*_ score. Point colour indicates the prediction accuracy tier—blue, high; amber, medium; red, low—and the shaded bands and dashed horizontal lines mark the LDDT_*pli*_ thresholds at 0.4 and 0.2. Marker shape indicates ligand chemistry: circle, nucleotide; square, rigid ligand; triangle, flexible cofactor; diamond, small metabolite. Labels, in neutral grey, identify only the ligands discussed in the text. The high tier comprises 21 AGS, 19 ADP, 9 Mg^2+^, 2 CoA, 2 DW0, 1 ATP, 1 sulphate and 1 acetate; the medium tier 4 Mg^2+^, 2 SAH and 1 ATP; and the low tier 6 Mg^2+^. Accuracy is highest for nucleotides, 41 of 42 of which fall in the high tier, whereas the rigid-ligand class—almost entirely Mg^2+^—spans all three tiers and accounts for every low-tier instance, reflecting the difficulty of placing small, diffusely coordinated metal ions. Tiers are defined on LDDT_*pli*_ and are independent of the BDT and MCC values reported in Table 1.

Flexible co-factors (CoA, SAH, and ATP) showed greater variability due to size and conformational flexibility, yet FunFOLD5 consistently recovered core anchoring regions, typically the adenosyl or adenine moiety. Approximately half of the predictions were of high quality, with most remaining in the medium tier and very few low-tier cases. This indicates robust identification of the correct binding pocket and global ligand orientation, even when residue-level precision is limited by ligand flexibility (Fig. 2 and Fig. 1, available as supplementary data at *Bioinformatics* online).

Small metabolites were also predicted with high overall accuracy. Acetate consistently yielded high-quality predictions due to its compact, well-constrained binding mode. DW0, representing weakly coordinated water-like ligands, also produced predominantly high-tier predictions, indicating that FunFOLD5 can localise plausible binding environments even without tightly defined pockets. The few lower-tier cases reflect intrinsic ligand ambiguity rather than methodological limitations (Fig. 2 and Fig. 1, available as supplementary data at *Bioinformatics* online).

Overall, FunFOLD5 demonstrated broad, chemically generalised performance, excelling in ligand classes with conserved or rigid structural motifs while also maintaining robust prediction quality for flexible cofactors and diffuse small molecules. With 81.2% of all predictions classified as high quality, FunFOLD5 consistently delivers accurate residue-level binding-site descriptions, even in challenging ligand environments. The limited number of medium- and low-tier outcomes reinforces that FunFOLD5 is a stable, dependable, and biologically interpretable method for ligand-binding site prediction, highly suited to structural genomics, function annotation, and CASP-level benchmarking (Fig. 2 and Fig. 1, available as supplementary data at *Bioinformatics* online).

### 3.2 Evaluating the impact of FunFOLD5’s local targeted protocol on binding-site identification

To quantify the specific contribution of FunFOLD5’s local targeted protocol, we compared predictions from the global-only workflow used in FunFOLD4 with those obtained from FunFOLD5’s combined global-and-local approach. The global protocol used in FunFOLD4 relies on whole-structure template similarity, which can miss locally conserved pocket features, position ligands inaccurately, or overlook flexible loop residues that define the functional binding environment. In contrast, FunFOLD5 adds a local targeted protocol that concentrates the search around the initially predicted pocket, improving identification of binding-site residues, ligand positioning, detection of specific ligand interactions, and agreement with conserved functional or catalytic motifs. This comparison, therefore, assesses several key aspects of binding-site prediction:

1. residue-level recall and precision,
2. ligand pose accuracy,
3. identification of specific or more chemically appropriate ligands, and
4. correspondence with known mechanistic residues.

In the previous section, we presented a comprehensive benchmarking of FunFOLD5 on CASP15 and CASP16 targets, as summarised in Table 1, available as supplementary data at *Bioinformatics* online. Since the global and local protocols work together within FunFOLD5, this section presents detailed case studies on a selected set of targets, chosen to isolate and highlight what the local targeted protocol specifically contributes. To avoid redundancy, a representative subset was selected for targets containing multiple copies of the same ligand. Template searches were restricted to structures released before May 2022 (the start of CASP15) to exclude *post hoc* structural information. CASP16 targets were excluded, as their predominantly novel folds provide limited template coverage and are, therefore, less suitable for evaluating the added value of the local targeted protocol.

The global-only workflow evaluated here is the FunFOLD4 protocol. FunFOLD4 identifies templates by whole-structure similarity alone, whereas FunFOLD5 applies the identical global pass and then adds the local targeted stage. The comparison below is therefore a controlled ablation against the previous version of the method, in which input models, template date restriction, and scoring protocol are held constant and only the local protocol varies. No comparison against third-party template-based servers is presented, since none participated in the CASP15 or CASP16 ligand categories and retrospective submission would not preserve blind conditions.

To quantitatively compare binding-site predictions from the global-only and local targeted protocols, we used the Matthews correlation coefficient (MCC) and the Binding-site Distance Test (BDT) score (Roche *et al.* 2010), which were formally adopted in CASP function prediction and CAMEO LB assessments. The MCC provides a strict residue-level classification metric but does not account for the 3D proximity of predicted residues to the true binding site, penalising near-miss predictions similarly to random ones. The BDT score complements the MCC by explicitly incorporating 3D distance information, producing continuous scores between 0 and 1 that reflect spatial agreement with the observed binding site. Used together, both the MCC and BDT provide a complementary and robust evaluation framework, enabling direct, quantitative comparison of binding-site quality and clear assessment of the improvements achieved by the local targeted protocol over the global approach.

A clear advantage of FunFOLD5’s combined approach is demonstrated for CASP15 target T1158v4, an ATP-binding protein whose experimental structure (PDB ID: 8swn) contains ATP but no BeF_x_ species. The global protocol alone, as used in FunFOLD4, recovers only residues 446–448, yielding a poorly defined pocket and a mispositioned ATP outside the catalytic centre, reflected by low accuracy (BDT = 0.156; MCC = 0.156; Table 1). By adding the local targeted protocol, FunFOLD5 identifies a more complete nucleotide-binding cleft, additionally recovering residues 450–453, 480, and 592 and enabling accurate ATP placement consistent with the observed structure (Fig. 3), which substantially improves prediction accuracy (BDT = 0.524, MCC = 0.585; Table 1).

**Figure 3:**
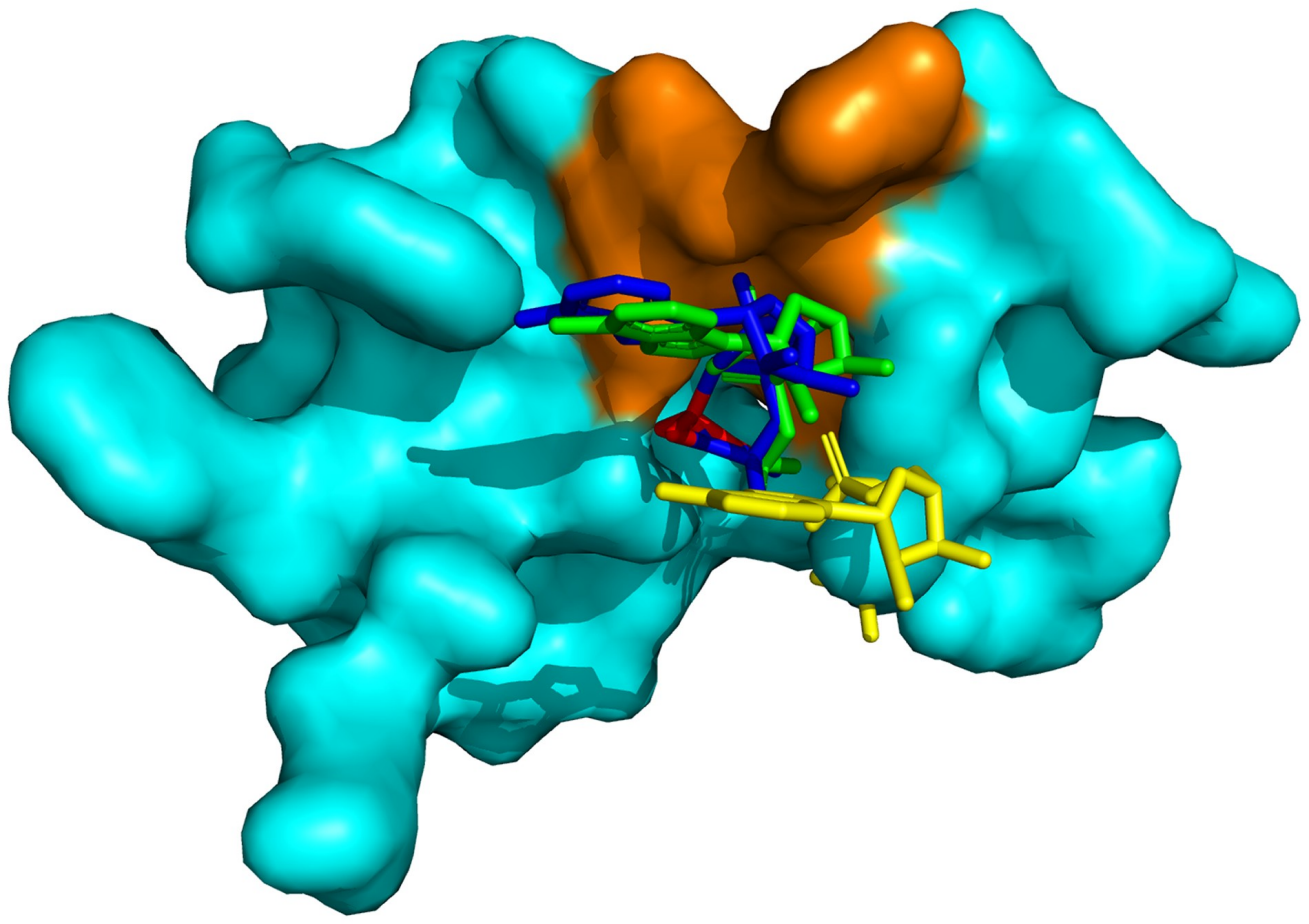
Improved binding-site prediction for T1158v4 using the local targeted protocol. Cyan shows the surface view of the pocket common to both protocols; orange highlights the specific pocket region identified only by FunFOLD5’s local targeted protocol. ATP is shown as sticks: blue (local targeted), green (experimental 8swn), and yellow (global). The FunFOLD4 protocol identifies only a limited subset of binding residues (446–448), resulting in low agreement with the experimental binding site (BDT = 0.156, MCC = 0.156; Table 1). In contrast, FunFOLD5’s local targeted protocol recovers specific functionally relevant residues (447, 448, 450–453, 480, and 592), producing an ATP pose that more closely matches the observed structure (BDT = 0.524, MCC = 0.585; Table 1). A BeF_x_ molecule predicted only by FunFOLD5’s local targeted protocol is shown in red sticks. BeF_x_ is absent from 8swn, so this prediction is excluded from all reported accuracy scores; it was transferred from the locally retrieved template 6yufC, in which ADP·BeF_3_ is experimentally resolved. As a known *γ*-phosphate transition-state analogue, its placement indicates a pocket geometry resembling a catalytically competent state (Cho *et al.* 2001, Kagawa *et al.* 2004, Danko and Suzuki 2016).

FunFOLD5’s local targeted protocol additionally predicts a BeF_x_ species at this site. BeF_x_ is absent from the experimental structure 8swn, so this prediction cannot be assessed against the reference and is excluded from all accuracy statistics reported here. It was transferred from a locally retrieved template, 6yufC, in which ADP·BeF_3_ is experimentally resolved, and which the global protocol did not retrieve. BeF_x_ is a well-established *γ*-phosphate transition-state analogue that stabilises catalytically competent conformations of ATPases (Cho *et al.* 2001, Kagawa *et al.* 2004, Danko and Suzuki 2016), so its placement here indicates that the locally refined pocket resembles a phosphate-transfer-competent state rather than the ground-state geometry captured in the deposited structure (Fig. 3). We present this as an illustration rather than a validated result, but it points to a broader use for the local protocol: because templates are retrieved on pocket similarity rather than global fold, the method can identify ligands that suggest specific catalytic or conformational states, which is information that global template transfer and single-pose ligand predictors may not provide. Systematic assessment of this capability would require benchmarks containing state-specific ligands, and is left for future work.

A similarly clear advantage from adding the local targeted protocol is observed for the Mg^2+^ site in CASP15 target T1158v4. In the experimental structure 8swn, Mg^2+^ is coordinated by residues 452 and 480, forming a key component of the ATP-dependent catalytic machinery. The global protocol alone, as used in FunFOLD4, predicts residues 447 and 480, partially overlapping with the true site but missing the critical coordinating residue 452, giving only moderate agreement (BDT = 0.596, MCC = 0.499; Table 1). With the local targeted protocol, FunFOLD5 correctly recovers both coordinating residues (452 and 480), yielding perfect agreement with the experimental site (BDT = 1.000, MCC = 1.000; Table 1). Together with the ATP and BeF_x_ results, this example demonstrates that the FunFOLD5 local protocol is particularly effective at resolving tightly constrained catalytic features—such as metal coordination—that the global protocol captures only partially, providing a more accurate and mechanistically faithful representation of the active site.

For each CASP target, experimentally observed binding residues (derived from the ligand-bound PDB structure) are compared with binding residues identified by FunFOLD4 global (first-round) and local targeted FunFOLD5 protocols. Binding-site prediction accuracy is quantified using the BDT and MCC scores, where higher values indicate better agreement with the experimental binding site. Residues listed under the FunFOLD5 local protocol include specific or refined binding-site positions that are not recovered by the global protocol, highlighting regions revealed through focused local refinement. Targets include nucleotide-, metal-, and small-molecule-binding proteins, enabling direct assessment of how local targeting improves residue-level recovery and spatial accuracy across different ligand types.

Beyond T1158v4, FunFOLD5’s local targeted protocol consistently improves residue-level binding-site identification across other CASP15 targets, compared with the global protocol used in FunFOLD4 (Table 1).

For T1124 (SAH), the FunFOLD4 global protocol predicts a dispersed and partially misaligned site (BDT = 0.604, MCC = 0.479), missing several residues that define the core SAH-binding environment (Fig. 3, available as supplementary data at *Bioinformatics* online). The FunFOLD5 local protocol recovers specific key residues, notably 167, 204, and 229, which are present in the experimental structure and refine the pocket towards the conserved adenine- and ribose-interacting region characteristic of SAM/SAH-dependent enzymes. Although the BDT score decreases slightly (0.422), the MCC increases to 0.533, indicating improved residue-level agreement and a more biologically meaningful representation of the SAH-binding site (Table 1). A second SAH instance shows the same effect more clearly. The FunFOLD4 global protocol commits to a compact but incomplete set (BDT = 0.556; MCC = 0.736), omitting the adenine-edge residues. The FunFOLD5 local protocol recovers 167, 203, 225, and 229—all present in the experimental structure—while discarding only residue 170, improving both metrics (BDT = 0.722; MCC = 0.843; Table 1). Unlike the first instance, here the sharper residue set improves spatial as well as residue-level agreement.

For CASP15 target T1127v2 (CoA; PDB ID: 8xbp), the FunFOLD5 local targeted protocol provides a clear improvement over the FunFOLD4 global approach (Table 1). The FunFOLD4 global protocol predicts a partially correct CoA-binding site with moderate accuracy (BDT = 0.772; MCC = 0.542) but fails to fully capture residues in the adenine- and phosphate-interacting region, resulting in a fragmented pocket (Fig. 4, available as supplementary data at *Bioinformatics* online). In contrast, the FunFOLD5 local protocol recovers specific residues, notably 180, 181, and 184, which define the canonical CoA-binding groove. Incorporating these residues refines pocket geometry and ligand placement, yielding substantially higher accuracy (BDT = 0.896; MCC = 0.820; Table 1).

For CASP15 target T1170 (ADP; PDB ID: 7pbr), the FunFOLD5 local targeted protocol consistently refines the FunFOLD4 global prediction (Table 1). The FunFOLD4 global protocol identifies a broadly correct ADP-binding site (BDT = 0.824; MCC = 0.653) but includes non-observed residues (e.g. 163) and omits residues at the N-terminal edge of the pocket. The FunFOLD5 local protocol sharpens site definition by recovering specific residues, notably 6 and 7, while excluding non-observed positions, resulting in a more specific and biologically coherent pocket (Fig. 5, available as supplementary data at *Bioinformatics* online). This refinement improves both spatial and residue-level agreement (BDT = 0.882; MCC = 0.740; Table 1). Two further ligand instances for CASP15 target T1170 [ADP (2) and AGS (1); PDB ID: 7pbr] show the same refinement in a higher-accuracy regime (Table 1). For ADP (2), the FunFOLD4 global protocol identifies a broadly correct site (BDT = 0.870; MCC = 0.812) but omits residue 6 at the N-terminal edge of the nucleotide pocket; the FunFOLD5 local protocol recovers this residue while retaining the remainder of the site, improving both metrics (BDT = 0.878; MCC = 0.860; Table 1). The same applies to AGS (1), where the global protocol scores BDT = 0.846 and MCC = 0.703 and the local protocol, again by recovering residue 6, reaches BDT = 0.856 and MCC = 0.751 (Table 1). The gains are modest because little of the pocket was missing, but they are in the same direction as for the more poorly defined sites.

For CASP15 target H1171 [ADP (1), AGS (1) and Mg^2+^ (2); PDB ID: 7pbl], a RuvB–RuvA complex, the FunFOLD4 global protocol already predicts the shared nucleotide site accurately [ADP (1), BDT = 0.867, MCC = 0.876; AGS (1), BDT = 0.826, MCC = 0.808] but includes residue 45, which is not observed in contact with any of the bound ligands. Rather than recovering additional positions, the FunFOLD5 local protocol here refines the site by excluding this non-observed residue while retaining all correctly predicted positions (6, 7, 13, 14, 46–52, 95, 166, 174, 202, 203, and 206), improving both metrics for all three ligands [ADP (1), BDT = 0.896, MCC = 0.903; AGS (1), BDT = 0.853, MCC = 0.833; Mg^2+^ (2), BDT = 0.417, MCC = 0.335; Table 1]. The Mg^2+^ (2) site scores low under both protocols because only two residues, 51 and 203, are observed in contact with the ion, so a pocket-scale prediction is penalised heavily regardless of its quality.

For CASP15 target H1172 [ADP (1) and AGS (2); PDB ID: 7pbp], which contains the same RuvB nucleotide site in a different subunit arrangement, the FunFOLD4 global protocol again provides a broadly correct prediction [ADP (1), BDT = 0.811, MCC = 0.679; AGS (2), BDT = 0.832, MCC = 0.695] but omits residue 6. The FunFOLD5 local protocol recovers this residue, improving both metrics for both ligands [ADP (1), BDT = 0.822, MCC = 0.727; AGS (2), BDT = 0.842, MCC = 0.738; Table 1]. As for T1170 and H1171, the gain is small in absolute terms, reflecting the limited scope for improvement when the global prediction is already close to the experimental site.

For CASP15 target T1188 (DW0; PDB ID: 8c6z), both the FunFOLD4 global protocol and the FunFOLD5 local targeted protocol show limited agreement with the experimentally observed binding site (Table 1). The DW0 site is small, involving only a few residues (120, 166, 172, 247, and 252), making predictions highly sensitive to residue-level errors. The FunFOLD4 global protocol predicts a large, diffuse residue set that is largely spatially inconsistent with the observed site, resulting in low accuracy (BDT = 0.243; MCC = −0.016; Table 1). The FunFOLD5 local protocol produces a more focused residue set (154, 156, 215, and 216) but still fails to recover the full binding site, yielding persistently lower accuracy (BDT = 0.078; MCC = −0.008; Table 1). Neither protocol recovers any observed binding residue, so the marginally negative MCC values reflect an absence of overlap rather than anti-correlation. DW0 is a synthetic ligand with no close analogue among biologically annotated templates, so clustering is driven by chemically unrelated template ligands in a different pocket; BDT, which measures spatial proximity rather than residue-number agreement, remains informative where MCC cannot discriminate. This case marks a boundary of applicability: template-based transfer degrades sharply for small, synthetic, or weakly conserved ligands.

Notably, the predicted DW0 binding poses from both protocols are spatially separated from one another and from the experimental ligand position, indicating a lack of convergence towards a single coherent binding pocket. This dispersion suggests that suitable ligand-bound templates for DW0 may be sparse or absent, limiting the ability of the template-based approach to correctly localise the binding site. Together, the small size of the binding site, incomplete residue recovery by the FunFOLD5 local protocol, and the lack of strong template signals likely contribute to the low BDT and MCC scores observed for this target (Fig. 6, available as supplementary data at *Bioinformatics* online). This contrasts with the substantial improvements achieved for larger, more structurally constrained nucleotide- and metal-binding sites, where local refinement is more effective.

Across the 14 ligand instances in Table 1, spanning seven CASP15 targets and covering co-factors, nucleotides, nucleotide analogues, and metal ions, the FunFOLD5 local targeted protocol improved residue-level agreement in every case (MCC increased in 14/14; mean ΔMCC +0.122; Wilcoxon signed-rank *P *= 0.001), and spatial agreement in 12 (mean ΔBDT +0.063). Gains were largest where the global protocol defined the site only partially, as for T1158v4 (ATP, ΔBDT +0.368; Mg^2+^, +0.404). More informative are the RuvB targets T1170, H1171, and H1172, where the global protocol already recovers the nucleotide sites accurately (BDT 0.811–0.870, MCC 0.653–0.876) and any further gain must come from single-residue corrections; even here every instance improved on both metrics (Table 1). The effect is therefore consistent regardless of the starting accuracy and across most of the ligand chemistries examined, indicating that local refinement systematically sharpens binding-site definition rather than correcting only poor predictions.

## 4 Conclusions

Template-based methods remain an essential component of protein–ligand interaction prediction, offering a powerful complement to modern deep learning approaches. By transferring experimentally validated structural information, they provide biologically grounded priors, such as ligand identity, binding-site geometry, and conserved chemical environments, which enhance interpretability and generalise to proteins beyond the scope of training data. FunFOLD5 advances protein–ligand interaction prediction by combining rapid structural template retrieval with a local targeted protocol that refines the protein ligand interactions. This local approach focuses on pocket-level similarity rather than global fold alignment, enabling more accurate recovery of ligand-binding residues, functional conformations, and chemically relevant ligands. In the CASP15 and CASP16 benchmarks, FunFOLD5 demonstrated strong performance across diverse ligand types, particularly for rigid and small-molecule ligands, where the FunFOLD5 local protocol improved pose accuracy and mechanistic relevance. It also successfully identified binding sites overlooked by global-only methods, including those in experimental structures. These results highlight the value of local structural context in predicting biologically meaningful interactions. As structure prediction continues to evolve, FunFOLD5 offers a practical and interpretable framework for functional annotation, with particular utility for uncharacterised or engineered proteins. These capabilities are especially valuable for structural genomics, enzyme function annotation, and protein design, where predicting the correct ligand type and its binding mode is key to inferring biological function. FunFOLD5 also provides transferable insights that can constrain and inform machine learning models, supporting the identification of multiple or allosteric sites and improving prediction robustness in data-limited settings. Looking forward, integrating FunFOLD5 with next-generation generative models promises to enhance both accuracy and scope, reinforcing the value of hybrid approaches for comprehensive, interpretable, and functionally meaningful protein–ligand interaction modelling.

## Supplementary Material

btag762_Supplementary_Data

## Data Availability

The FunFOLD5 pipeline is available at https://hub.docker.com/r/radiyaman/funfold5_template. Experimental structures used for evaluation are available from the Protein Data Bank under the accession codes given in Table 1 and Table 1, available as supplementary data at *Bioinformatics* online.
