# Supplementary material for: FunFOLD5: combining localised templates and structural homology for accurate predictions of biologically relevant protein–ligand interactions
Source: Bioinformatics. 2026 Oct 10;42(10):btag762. doi: 10.1093/bioinformatics/btag762 (PMC13711230; doi:10.1093/bioinformatics/btag762)
Supplement: Supplementary file 1 [file btag762_supplementary_data.docx]

**Supplementary Table 1. Summary of FunFOLD5 ligand-binding predictions across CASP15 and CASP16 targets.** For each ligand instance, the table reports the CASP target ID, ligand identity, associated PDB structure, chemistry category, CASP ranking, LDDTpli score, ligand RMSD (Å), and assigned accuracy tier. Ligand-binding accuracy was evaluated using LDDTpli and classified as High (≥ 0.4), Medium (0.2–0.4), or Low (< 0.2). The dataset spans rigid ligands (e.g. SO₄²⁻, Mg²⁺), small metabolites (ACT, DW0), flexible cofactors (SAH, COA), and nucleotides (ATP, ADP, AGS), enabling comparative assessment of FunFOLD5 performance across ligand chemistries, binding-site architectures, and benchmark rankings.

| CASP16 |  |  |  |  |  |  |  |
| --- | --- | --- | --- | --- | --- | --- | --- |
| CASP ID | Ligand | PDB ID | Chemistry Category | Ranking | LDDTpli | RMSD (Å) | Tier |
| L3102 | 1, SO4 |  | Rigid ligand | 9 | 0.688 | 2.28 | High |
| L3132 | 3, ACT |  | Small metabolite | 89 | 0.585 | 3.402 | High |
| CASP15 |  |  |  |  |  |  |  |
| CASP ID | Ligand | PDB ID | Chemistry Category | Ranking | LDDTpli | RMSD (Å) | Tier |
| T1124 | 1, SAH | 7ux8 | Flexible cofactor | 305 | 0.293 | 9.466 | Medium |
| T1124 | 2, SAH | 7ux8 | Flexible cofactor | 332 | 0.318 | 8.882 | Medium |
| T1127v2 | 1, COA | 8xbp | Flexible cofactor | 137 | 0.462 | 6.161 | High |
| T1127v2 | 2, COA | 8xbp | Flexible cofactor | 110 | 0.577 | 6.069 | High |
| T1158v4 | 1, ATP | 8swn | Nucleotide | 126 | 0.2 | 9.065 | Medium |
| T1158v4 | 2, ATP | 8swn | Nucleotide | 98 | 0.472 | 5.774 | High |
| T1158v4 | 3, MG | 8swn | Rigid ligand | 7 | 0.839 | 1.995 | High |
| T1158v4 | 4, MG | 8swn | Rigid ligand | 90 | 0.55 | 3.35 | High |
| T1170 | 1, ADP | 7pbr | Nucleotide | 31 | 0.894 | 0.807 | High |
| T1170 | 2, ADP | 7pbr | Nucleotide | 21 | 0.881 | 0.845 | High |
| T1170 | 4, AGS | 7pbr | Nucleotide | 15 | 0.691 | 2.055 | High |
| T1170 | 5, AGS | 7pbr | Nucleotide | 18 | 0.541 | 3.231 | High |
| T1170 | 6, AGS | 7pbr | Nucleotide | 11 | 0.754 | 1.706 | High |
| T1170 | 10, AGS | 7pbr | Nucleotide | 4 | 0.828 | 1.286 | High |
| T1188 | 1, DW0 | 8c6z | Small metabolite | 67 | 0.426 | 4.652 | High |
| T1188 | 2, DW0 | 8c6z | Small metabolite | 35 | 0.601 | 6.391 | High |
| H1171v1 | 1, ADP | 7pbl | Nucleotide | 4 | 0.906 | 0.795 | High |
| H1171v1 | 4, AGS | 7pbl | Nucleotide | 6 | 0.744 | 2.09 | High |
| H1171v1 | 5, AGS | 7pbl | Nucleotide | 12 | 0.748 | 2.219 | High |
| H1171v1 | 6, AGS | 7pbl | Nucleotide | 1 | 0.881 | 1.321 | High |
| H1171v1 | 7, MG | 7pbl | Rigid ligand | 50 | 0.325 | 8.105 | Medium |
| H1171v1 | 10, MG | 7pbl | Rigid ligand | 26 | 0.278 | 8.17 | Medium |
| H1171v1 | 11, MG | 7pbl | Rigid ligand | 35 | 0 | 9.915 | Low |
| H1171v2 | 1, ADP | 7pbl | Nucleotide | 1 | 0.895 | 0.941 | High |
| H1171v2 | 2, ADP | 7pbl | Nucleotide | 20 | 0.867 | 0.997 | High |
| H1171v2 | 3, ADP | 7pbl | Nucleotide | 24 | 0.887 | 0.927 | High |
| H1171v2 | 4, AGS | 7pbl | Nucleotide | 13 | 0.743 | 2.049 | High |
| H1171v2 | 5, AGS | 7pbl | Nucleotide | 14 | 0.719 | 2.111 | High |
| H1171v2 | 6, AGS | 7pbl | Nucleotide | 4 | 0.82 | 1.175 | High |
| H1171v2 | 7, MG | 7pbl | Rigid ligand | 77 | 0 | 9.811 | Low |
| H1171v2 | 8, MG | 7pbl | Rigid ligand | 68 | 0.306 | 8.257 | Medium |
| H1171v2 | 9, MG | 7pbl | Rigid ligand | 68 | 0 | 9.008 | Low |
| H1172v1 | 1, ADP | 7pbp | Nucleotide | 3 | 0.784 | 2.033 | High |
| H1172v1 | 2, ADP | 7pbp | Nucleotide | 1 | 0.905 | 0.769 | High |
| H1172v1 | 3, ADP | 7pbp | Nucleotide | 32 | 0.864 | 0.965 | High |
| H1172v1 | 4, AGS | 7pbp | Nucleotide | 8 | 0.795 | 1.218 | High |
| H1172v1 | 5, AGS | 7pbp | Nucleotide | 6 | 0.792 | 1.261 | High |
| H1172v1 | 6, AGS | 7pbp | Nucleotide | 1 | 0.82 | 1.175 | High |
| H1172v1 | 7, MG | 7pbp | Rigid ligand | 77 | 0.056 | 8.082 | Low |
| H1172v1 | 8, MG | 7pbp | Rigid ligand | 68 | 0 | 8.723 | Low |
| H1172v1 | 9, MG | 7pbp | Rigid ligand | 68 | 0.083 | 8.476 | Low |
| H1172v2 | 1, ADP | 7pbp | Nucleotide | 1 | 0.895 | 0.941 | High |
| H1172v2 | 2, ADP | 7pbp | Nucleotide | 20 | 0.867 | 0.997 | High |
| H1172v2 | 3, ADP | 7pbp | Nucleotide | 24 | 0.887 | 0.927 | High |
| H1172v2 | 4, AGS | 7pbp | Nucleotide | 8 | 0.806 | 1.233 | High |
| H1172v2 | 5, AGS | 7pbp | Nucleotide | 16 | 0.719 | 2.111 | High |
| H1172v2 | 6, AGS | 7pbp | Nucleotide | 2 | 0.837 | 1.149 | High |
| H1172v2 | 7, MG | 7pbp | Rigid ligand | 14 | 0.833 | 3.361 | High |
| H1172v2 | 8, MG | 7pbp | Rigid ligand | 31 | 0.778 | 2.728 | High |
| H1172v2 | 9, MG | 7pbp | Rigid ligand | 27 | 0.806 | 3.511 | High |
| H1172v3 | 1, ADP | 7pbp | Nucleotide | 30 | 0.883 | 0.936 | High |
| H1172v3 | 2, ADP | 7pbp | Nucleotide | 29 | 0.407 | 4.894 | High |
| H1172v3 | 3, ADP | 7pbp | Nucleotide | 3 | 0.875 | 1.225 | High |
| H1172v3 | 4, ADP | 7pbp | Nucleotide | 4 | 0.9 | 0.767 | High |
| H1172v3 | 5, AGS | 7pbp | Nucleotide | 7 | 0.799 | 1.168 | High |
| H1172v3 | 6, AGS | 7pbp | Nucleotide | 6 | 0.821 | 1.171 | High |
| H1172v3 | 7, MG | 7pbp | Rigid ligand | 40 | 0.7 | 2.934 | High |
| H1172v3 | 8, MG | 7pbp | Rigid ligand | 41 | 0.75 | 2.861 | High |
| H1172v4 | 1, ADP | 7pbp | Nucleotide | 25 | 0.894 | 0.832 | High |
| H1172v4 | 2, ADP | 7pbp | Nucleotide | 3 | 0.882 | 1.174 | High |
| H1172v4 | 3, ADP | 7pbp | Nucleotide | 30 | 0.882 | 0.852 | High |
| H1172v4 | 4, AGS | 7pbp | Nucleotide | 6 | 0.818 | 1.149 | High |
| H1172v4 | 5, AGS | 7pbp | Nucleotide | 7 | 0.816 | 1.149 | High |
| H1172v4 | 6, AGS | 7pbp | Nucleotide | 1 | 0.85 | 1.215 | High |
| H1172v4 | 7, MG | 7pbp | Rigid ligand | 128 | 0.306 | 6.22 | Medium |
| H1172v4 | 8, MG | 7pbp | Rigid ligand | 37 | 0.722 | 2.809 | High |
| H1172v4 | 9, MG | 7pbp | Rigid ligand | 22 | 0.792 | 3.333 | High |

**Supplementary Figure 1. FunFOLD5 ligand-binding prediction quality by tier and ligand category.** The stacked bars show FunFOLD5 performance across three LDDTpli tiers: 56 ligands in the high tier (81.2%), 7 in the medium tier (10.1%), and 6 in the low tier (8.7%), with colours representing ligand categories: blue for flexible cofactors, orange for nucleotides, green for rigid ligands, and red for small metabolites. In the high tier, nucleotides shown in orange dominate the distribution with 19 ADP, 21 AGS, and 1 ATP, reflecting consistently strong prediction accuracy. Rigid ligands shown in green add 9 Mg²⁺ and 1 sulfate, while flexible cofactors in blue contribute 2 CoA and small metabolites in red contribute acetate and two DW0 ligands. The medium tier consists of ATP and two SAH predictions and four Mg²⁺. The low tier contains six Mg²⁺, all shown in green, corresponding to weakly coordinated or solvent-exposed metal sites. The colour pattern and tier percentages highlight FunFOLD5’s strongest performance for nucleotides and well-defined rigid ligands, with reduced accuracy confined largely to ambiguous Mg²⁺ environments.

**Supplementary Figure 2. Improved Mg²⁺ binding-site prediction for CASP15 target T1158v4 using the FunFOLD5 local targeted protocol.** The binding pocket common to both the FunFOLD4 global and FunFOLD5 local protocols is shown in cyan surface view, while orange highlights the specific pocket region identified exclusively by the FunFOLD5 local targeted protocol. The Mg²⁺ ion is shown as a sphere: green for the experimentally observed ion in 8SWN, blue for the FunFOLD5 local targeted prediction, and yellow for the FunFOLD4 global prediction. The FunFOLD4 global protocol identifies only a partial coordination site, whereas the FunFOLD5 local targeted protocol correctly recovers the experimentally observed Mg²⁺-coordinating residues 452 and 480, resulting in perfect agreement with the native structure (BDT = 1.000; MCC = 1.000; Table 1). This illustrates how local refinement enables precise identification of tightly constrained metal-binding geometries that are incompletely captured by the FunFOLD4 global protocol.

**Supplementary Figure 3. Improved SAH binding-site prediction for CASP target T1124 using the FunFOLD5 local targeted protocol.**The binding pocket common to both the FunFOLD4 global and FunFOLD5 local protocols is shown in cyan surface view, while **orange** highlights the specific pocket region identified exclusively by the FunFOLD5 local targeted protocol. **SAH** is shown as sticks: **blue** for the FunFOLD5 local targeted prediction, **green** for the experimentally observed ligand in **7ux8**, and **yellow** for the FunFOLD4 global prediction. The FunFOLD4 global protocol predicts a dispersed set of binding residues, resulting in a less well-defined SAH-binding pocket (BDT = 0.604; MCC = 0.479; Table 1). In contrast, the FunFOLD5 local targeted protocol recovers specific residues, including **167, 204, and 229**, refining the pocket geometry and improving residue-level agreement with the experimental binding site (BDT = 0.422; MCC = 0.533; Table 1). This example illustrates how local targeting sharpens binding-site definition and improves correspondence with the native ligand-binding environment.

**Supplementary Figure 4. Improved CoA binding-site prediction for CASP15 target T1127v2 using the FunFOLD5 local targeted protocol.** The binding pocket common to both the FunFOLD4 global and FunFOLD5 local protocols is shown in cyan mesh view, while orange highlights the specific pocket region identified exclusively by the FunFOLD5 local targeted protocol. CoA is shown as sticks: blue for the FunFOLD5 local targeted prediction, green for the experimentally observed ligand in 8xbp, and yellow for the FunFOLD4 global prediction. The FunFOLD4 global protocol captures a partial CoA-binding site, yielding moderate agreement with the experimental structure (BDT = 0.772; MCC = 0.542; Table 1). In contrast, the FunFOLD5 local targeted protocol recovers specific residues—most notably 180, 181, and 184—which refine the adenine- and phosphate-interacting region of the CoA-binding pocket. This expanded and more coherent pocket results in substantial improvements in both spatial and residue-level accuracy (BDT = 0.896; MCC = 0.820; Table 1), demonstrating that local refinement markedly enhances identification of functionally relevant CoA-binding features.

**Supplementary Figure 5. Improved ADP binding-site prediction for CASP15 target T1170 using the FunFOLD5 local targeted protocol.** The binding pocket common to both the FunFOLD4 global and FunFOLD5 local protocols is shown in cyan surface view, while orange highlights the specific pocket region identified exclusively by the FunFOLD5 local targeted protocol. ADP is shown as sticks: blue for the FunFOLD5 local targeted prediction, green for the experimentally observed ligand in 7pbr, and yellow for the FunFOLD4 global prediction. The FunFOLD4 global protocol captures a broadly correct ADP-binding site but includes non-observed residues, yielding moderate agreement with experiment (BDT = 0.824; MCC = 0.653; Table 1). The FunFOLD5 local targeted protocol refines the pocket by recovering specific experimentally observed residues (including 6 and 7) and removing spurious contacts, resulting in improved spatial localisation and residue-level accuracy (BDT = 0.882; MCC = 0.740; Table 1).

**(A)	(B)**

**Supplementary Figure 6. Comparison of FunFOLD4 global and FunFOLD5 local targeted binding-site predictions for CASP15 target T1188 (DW0).**

(A) Close-up view of the FunFOLD5 local DW0-binding region. The binding pocket shared by both the FunFOLD4 global and FunFOLD5 local protocols is shown in cyan surface view. DW0 ligands are shown as sticks: blue for the FunFOLD5 local targeted prediction, yellow for the FunFOLD4 global prediction, and green for the experimentally observed ligand in PDB ID 8c6z. (B) Global structural context of the binding site. The full protein structure is shown as a magenta cartoon, with the predicted binding pocket overlaid in cyan, illustrating the shallow and weakly defined nature of the FunFOLD5 local DW0-binding region.

# Deployment and usage

FunFOLD5 is distributed as a fully Dockerised, template-based prediction pipeline, enabling reproducible execution without manual dependency installation. The containerised implementation (radiyaman/funfold5_template) packages the complete FunFOLD5-template workflow developed by the McGuffin Group and is publicly available via Docker Hub (Adiyaman, n.d.). Containerisation ensures consistent behaviour across systems and allows execution on standard CPU-only Linux machines, without GPU requirements or prior model generation.

The Docker image can be obtained from Docker Hub:

docker pull radiyaman/funfold5_template:latest

The container was developed and tested on Linux systems (Ubuntu 20.04) and requires only a functional Docker installation.

Execution is controlled via three environment variables:

- **WORKING_FOLDER**: directory where all outputs are written
- **MODEL_FILE**: input protein structure model in PDB format
- **BIOLIP_Directory**: path to the locally mounted BioLiP2 database

```
A typical execution command is:
docker run --rm \
  -e WORKING_FOLDER=/data/work \
  -e MODEL_FILE=T1214.pdb \
  -e BIOLIP_Directory=/data/biolip \
  -v /path/to/working_folder:/data/work \
  -v /path/to/BioLip2:/data/biolip \
  radiyaman/funfold5_template:latest \
  bash /FunFOLD5_files/run_FunFOLD5.sh
```

FunFOLD5 requires a local copy of the BioLiP2 database, which provides curated, biologically relevant protein–ligand interactions. BioLiP2 was obtained from the Zhang Group repository and mounted into the container at runtime. The full dataset can be downloaded using:

wget https://zhanggroup.org/BioLiP/download/download_all_sets.txt

perl download_all_sets.txt

All outputs are written to a FunFOLD5 subdirectory within the user-defined working folder and are designed to support structural inspection, functional annotation, and downstream comparative analyses. Key files are located in FunFOLD5/predictions, which contains all ligand-coordinate outputs used for binding-site interpretation.

**Key outputs include:**

- **Ligand and structure files**
  - *_ligands.pdb: all ligands transferred from template structures.
  - *_lig.pdb: ligands aggregated from all contributing templates.
  - *_lig2.pdb: ligands grouped by cluster, representing cluster-based classification of binding-site hypotheses.
  - *_lig3.pdb: the most central (centroid) ligand from the dominant cluster, representing the final predicted ligand pose.
     These files enable direct visual comparison of global versus locally refined ligand placements.
- **Template lists** (Foldseek_*.dat, *.m8), documenting the structural templates contributing to each predicted binding site and providing full transparency of template evidence.
- **Binding-site residue files** (bindingsite*.txt, Allbindingsite*.txt), reporting predicted binding residues for each site, optionally including neighbouring residues within 8 Å.
- **Summary and annotation files** (*_FN.txt, all_ligand_list.txt, all_ligand_summary_list.txt), consolidating predicted ligands, binding residues, GO annotations, clustering results, and template statistics.
- **Template statistics** (summary_3D_sequence_*.txt, summary_TM_sequence_*.txt), reporting alignment scores, E-values, and TM-scores to support assessment of template quality and relevance.
